# Nucleosome eviction along with H3K9ac deposition enhances Sox2 binding during human neuroectodermal commitment

**DOI:** 10.1038/cdd.2017.62

**Published:** 2017-05-05

**Authors:** Yanhua Du, Zhenping Liu, Xinkai Cao, Xiaolong Chen, Zhenyu Chen, Xiaobai Zhang, Xiaoqing Zhang, Cizhong Jiang

**Affiliations:** 1 Institute of Translational Research, Tongji Hospital, Tongji University, Shanghai 200065, China; 2 The School of Life Sciences and Technology, Shanghai Key Laboratory of Signaling and Disease Research, The Collaborative Innovation Center for Brain Science, Tongji University, Shanghai 200092, China; 3 Tongji University School of Medicine, Shanghai 200092, China

## Abstract

Neuroectoderm is an important neural precursor. However, chromatin remodeling and its epigenetic regulatory roles during the differentiation of human neuroectodermal cells (hNECs) from human embryonic stem cells (hESCs) remain largely unexplored. Here, we obtained hNECs through directed differentiation from hESCs, and determined chromatin states in the two cell types. Upon differentiation, H2A.Z-mediated nucleosome depletion leads to an open chromatin structure in promoters and upregulates expression of neuroectodermal genes. Increase in H3K9ac signals and decrease in H3K27me3 signals in promoters result in an active chromatin state and activate neuroectodermal genes. Conversely, decrease in H3K9ac signals and increase in H3K27me3 signals in promoters repress pluripotency genes. Moreover, H3K9ac signals facilitate the pluripotency factor Sox2 binding to target sites unique to hNECs. Knockdown of the acetyltransferase Kat2b erases H3K9ac signals, disrupts Sox2 binding, and fails the differentiation. Our results demonstrate a hierarchy of epigenetic regulation of gene expression during the differentiation of hNECs from hESCs through chromatin remodeling.

Chromatin remodeling offers the epigenetic basis for transcriptional regulation and has a pivotal role in many biological processes such as cell differentiation, embryonic development, and so on. Nucleosome is the fundamental repeating structural unit of chromatin.^1^ Cell differentiation involves extensive nucleosome reorganization that alters chromatin structure. Lineage-specified cells have a more condensed chromatin structure compared to pluripotency stem cells.^2^ Nucleosomes are evicted upstream near transcription start sties (TSSs) or in distant enhancers to activate genes required for lineage commitment during differentiation of stem cells.^3, 4, 5^ Genome-wide comparisons of chromatin structure between mammalian pluripotent stem cells and differentiated cell types reveal that regions of difference in nucleosome occupancy are enriched in the loci associated with pluripotency and differentiation.^6^ Thus, dynamics of nucleosome positioning is important to cell fate commitment.

Histone modifications (HMs) are another key impact factor for chromatin structure. HMs exert their epigenetic regulatory roles through changing chromatin state or recruiting non-histone proteins to chromatin.^7^ Global changes in HMs during multilineages differentiation of human embryonic stem cells (ESCs) have been reported in previous studies.^8, 9^ Comparisons between human pluripotent stem cells and donor fibroblasts identified lots of regions with differences in H3K4me3 and H3K27me3 signals.^10^ Profiling of HMs of mouse ESCs, neural progenitor cells (NPCs), and embryonic fibroblasts revealed that H3K4me3 and H3K27me3 effectively distinguish genes with different expression levels and reflect lineage potential.^11^ A recent HPLC-MS-based quantitative proteomics identified dozens of HM sites in mouse ESCs and NPCs and elaborated the effect of combinational HMs on the differentiation of mouse ESCs to NPCs.^12^ The above studies all mainly focused on profiling of HMs and the global changes from ESCs to NPCs. However, the role of HM changes in the differentiation largely remains unclear.

Transcription factors' (TFs) binding to the target sites is closely correlated with HMs for a precise regulation of gene expression. Some TFs contain a protein domain that recognizes specific type of HM. Consequently, the TFs may have an affinity for the specific HM. For example, bromodomain PHD finger TF (BPTF) has a higher affinity to H3K4me3 than H3K4me2 and discriminates against H3K4me1.^13^ It was reported that the core pluripotency TF Oct4 binds to the target sites with primed epigenetic signatures during mouse somatic cell reprogramming.^14^ Motifs of distinct TFs are enriched in the regions with stage-specific transition during neural differentiation.^15^ However, it is unclear which HM(s) have the dominant role and how it regulates TFs' binding. It is also under debate at most time as whether HMs are causal or a consequence of TFs' binding.

Neuroectoderm specification is of major interest to developmental biology. Neuroectoderm (neuroepithelia) is a very important neural precursor. It develops into neural plate, providing the source of the central nervous system.^16^ Moreover, neuroectoderm has potentials to differentiate into neurons, astrocytes, and oligodendrocytes. Therefore, the differentiation of ESCs to neuroectoderm is of great significance for understanding of neural disease, for example, primitive neuroectodermal tumors. Epigenetic regulation has a critical role in the differentiation. For example, a subset of poised enhancers gained active chromatin signatures (H3K27ac) during differentiation of hESCs to neuroepithelium.^17^ However, to date, the epigenetic regulatory mechanisms underlying neuroectoderm specification remains elusive.

To unveil the patterns and roles of chromatin remodeling during the differentiation of human ESCs (hESCs) to human neuroectodermal cells (hNECs), we employed the differentiation method of hESCs to a synchronized population of hNECs.^18, 19^ This differentiation process resembles *in vivo* neural development and serves a model to study the mechanisms underlying human neuroectoderm specification.^20^ For example, Pax6 was identified as a transcriptional determinant of human neuroectoderm through this neural differentiation tool.^21^ In this study, we profiled transcriptome, nucleosome occupancy, core histone modifications, and genomic bindings of TF Sox2 for hESCs and hNECs. The integrative analyses of these data show that extensive nucleosome repositioning and histone modification changes occur upon differentiation. H2A.Z-mediated nucleosome disassembly in promoters leads to an open chromatin structure to activate transcription of neuroectodermal genes. Moreover, H3K9ac signals deposited by acetyltransferase Kat2b are essential to the differentiation through enhancing recruitment neuroectodermal factors Sox2 and Pax6 to their target sites.

## Results

### Expression profiles upon differentiation

**Figure 1:**
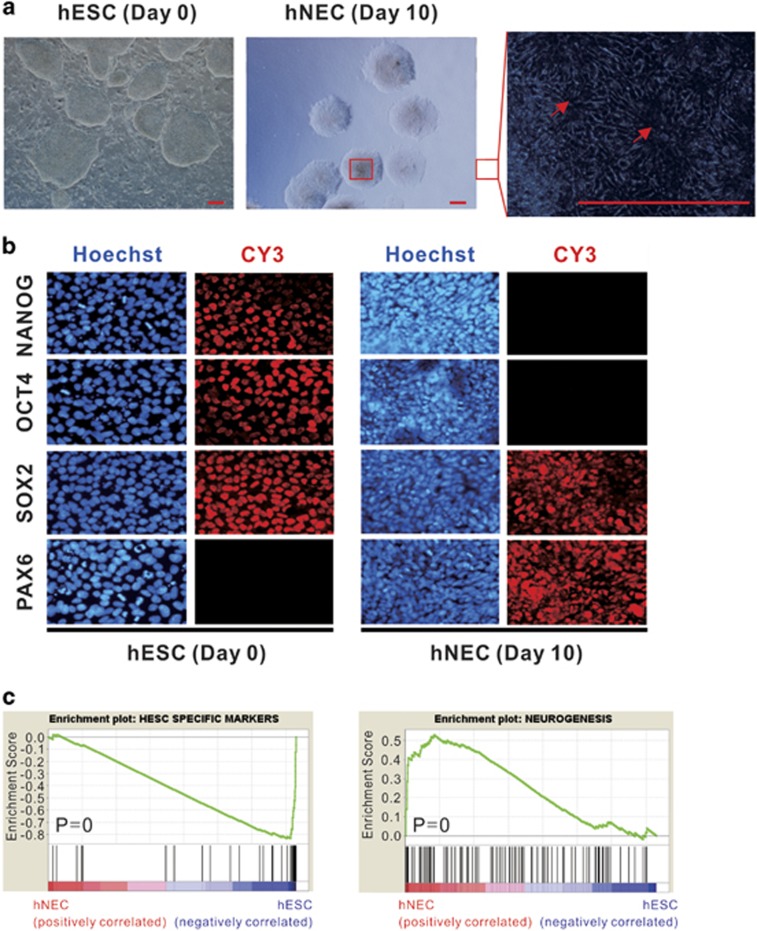
Expression profiles along hESCs differentiating to hNECs. (**a**) Microscopy images showing cell morphology changes between hESCs and hNECs. The right panel is an enlargement of hNEC colony marked by a red box. Arrows indicating that the differentiated hNECs are organized in rosettes. Scale bars, 100 *μ*m. (**b**) Immunofluorescent staining showing expression of TFs Nanog, Oct4, Sox2 and Pax6 in hESCs and hNECs, respectively. (**c**) GSEA results: hESC-specific genes are downregulated in hNECs whereas neurogenesis genes are upregulated

We employed a chemically defined protocol to make directed differentiation of hESCs to nearly pure hNECs.^18, 19^ The synchronized population of hNECs have a characteristic of rosette formation that is morphologically distinct from hESCs (Figure 1a). The qPCR results show that the mRNA levels of core pluripotency genes Oct4 (also known as Pou5f1) and Nanog are significantly decreased in hNECs whereas the hNEC determinant factor Pax6 is opposite (Supplementary Figure S1a). Immunofluorescence assay confirmed the similar results at protein levels (Figure 1b). Of note, Sox2 has a retention of high protein levels in both hESCs and hNECs. This is consistent with the critical roles of Sox2 in both ESCs and NPCs.^22, 23^ We'll discuss more about the functions of Sox2 on the differentiation of hNECs hereinafter.

Gene set enrichment analysis (GSEA)^24^ revealed that hESC-specific marker genes are significantly repressed during the differentiation, whereas the genes associated with neurogenesis and central nervous system development are significantly upregulated (Figure 1c and Supplementary Figure S1b). The profiling of changes in global gene expression identified 2477 significantly differentially expressed genes (DEGs), 1033 upregulated and 1444 downregulated in hNECs (Supplementary Figure 1c). Gene ontology (GO) analysis found that the upregulated DEGs are enriched for terms related to cell differentiation and neurogenesis (Supplementary Figure 1d). Taken together, the expression profiles consist with the corresponding cell identities. The results confirmed the purity of differentiated hNECs at the molecular levels.

### H2A.Z-mediated nucleosome eviction

**Figure 2:**
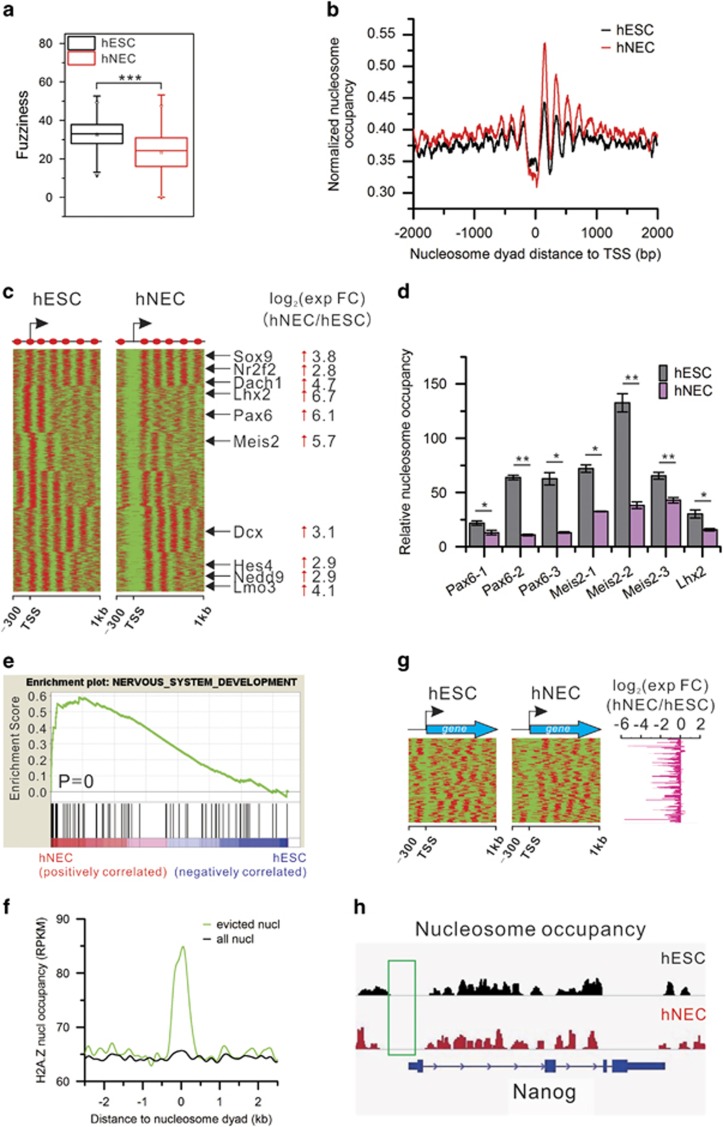
Nucleosome remodeling upon the differentiation. (**a**) Nucleosome fuzziness is significantly decreased in hNECs. (*P*-value=2.2e−16, Student's *t*-test). (**b**) Composite nucleosome distribution around TSS. (**c**) NDR formation in hNECs around TSS of a set of genes. Representative neuroectodermal genes are listed in the middle whose expression change folds are indicated at right. Arrows indicate upregulation in hNECs. (**d**) ChIP-qPCR confirmation of decreased nucleosome occupancy in NDRs of the selected neuroectodermal genes. Error bars represent SEM. (*P<0.05, **P<0.01, Student's *t*-test). (**e**) GSEA showing that nervous system development genes are enriched in the genes in **c** with an NDR in their promoters and upregulated in hNECs. (**f**) Nucleosomes evicted in hNECs are abundant with H2A.Z nucleosomes. (**g**) NDRs remain around TSS of pluripotency genes whose expression levels are greatly decreased in hNECs. (**h**) Track view of NDRs around TSS of pluripotency TF Nanog maintained in both hESCs and hNECs. Green box indicates the NDRs

We scanned the genome with a 10-kb window and calculated nucleosome occupancy for each window. Nucleosome occupancy of the biological replicates is highly reproducible (Supplementary Figure S2a). There occur global extensive changes in nucleosome occupancy in the differentiation with significant occupancy increase in most genomic regions in hNECs (Supplementary Figure S2b). We obtained the same results with different offsets and window sizes (data not shown here). Moreover, the nucleosome positioning becomes more fixed in hNECs than in hESCs (Figure 2a). As a result, chromatin structure becomes more condensed and stable in hNECs. This is consistent with the chromatin structures in pluripotent stem cells and differentiated cells, respectively.^2^

We next predicted nucleosome positions using nucleosome prediction tool GeneTrack.^25^ Comparisons found that only 1% of nucleosomes are fixed without changing their positions upon differentiation, 6% are disassembled or reassembled, and the rest shift. Further analysis show that nucleosome loss is enriched in promoters (Supplementary Figure S2c). This finding suggests that there are extensive nucleosome repositioning during the differentiation.

Nucleosome organization in promoters has a critical role in regulation of gene expression.^26^ There exists a canonical nucleosome organization of −1, nucleosome depletion region (NDR), +1, +2, *et al.* nucleosomes around TSS in both cell types. The average nucleosome occupancy in promoters is lower in hESCs than hNECs. Surprisingly, there is a small peak in the NDR in hESCs indicating that NDRs of some genes are occupied by nucleosomes in hESCs but free of nucleosomes in hNECs (Figure 2b). The nucleosome occupancy in the NDRs (−150–50 bp of TSS) is negatively correlated with gene expression levels (Supplementary Figure S2d). This suggests the key roles of NDRs in regulating differentiation.

To address the role of NDR formation in the differentiation of hNECs, we identified genes containing an NDR in hNECs that is occupied by a nucleosome in hESCs. Nucleosome arrangement around TSS of these genes is similar in both cell types except for an occupied NDR in hESCs and an unoccupied NDR in hNECs (Figure 2c). Intriguingly, this group of genes include many neuroectodermal genes such as Lhx2, Pax6, Meis2 and so on. As a result, their expression levels are significantly increased. ChIP-qPCR results confirmed the significantly reduced nucleosome occupancy in the NDRs (Figure 2d). GSEA results further show that ‘nervous system development' gene set is activated upon differentiation (Figure 2e). Remarkably, H2A.Z nucleosomes are enriched on the evicted nucleosomes that form the NDRs in hNECs (Figure 2f). This suggests that H2A.Z nucleosomes mediate NDR formation in promoters of neuroectodermal genes to facilitate the differentiation of hNECs. As contrast, we collected pluripotency genes and examined nucleosome organization around TSS. Unexpectedly, NDRs retain unoccupied during the differentiation (Figures 2g and h; Supplementary Figure S2e). However, their expression levels are significantly decreased (Figure 2g). This indicates that other factors other than nucleosome occupancy in NDRs regulate their activity.

We further analyzed the distribution of evicted nucleosomes and H2A.Z nucleosomes around TSS. The results show that evicted nucleosomes are enriched in the canonical −1, NDR, +1, +2 nucleosomes. Consistently, H2A.Z nucleosomes are also enriched in this region (Supplementary Figure S2f). In addition, only 1.2% of evicted nucleosomes and 10.4% of H2A.Z nucleosomes are located around TSS, respectively. These findings suggest that nucleosome eviction is not restricted to the promoter regions on the genome scale.

### Establishment of a permissive chromatin state in promoters

**Figure 3:**
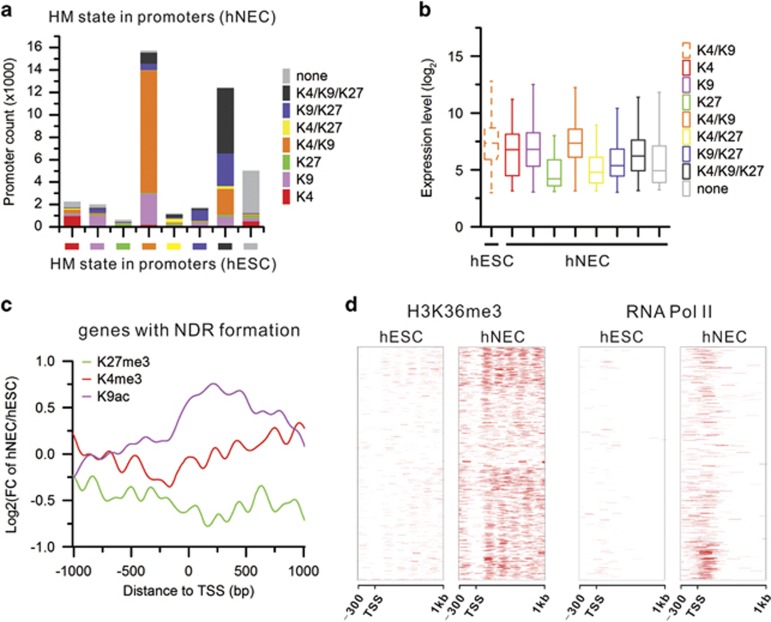
Establishment of a permissive chromatin state in the promoter regions. (**a**) Statistics summary of each category of promoters defined by histone modification mark(s) in hESCs changing into other categories in hNECs. (**b**) Changes in expression levels of genes with H3K4me3/H3K9ac in hESCs upon differentiation to hNECs. Gain of repressive H3K27me3 is associated with decreased expression. (**c**) Fold change of core histone modification signals in the promoters with NDR formation upon differentiation. (**d**) Increased H3K36me3 signals and RNA Pol II binding in the genes with NDR formation in the promoters upon differentiation

To uncover how HMs change and regulate gene expression during the differentiation, we generated genome-wide occupancy maps of core HMs using high-throughput ChIP-seq technology with high reproducibility (Supplementary Figure S3a). We next categorized promoters by HM marks. The majority of promoters are marked by H3K4me3/H3K9ac or H3K4me3/H3K9ac/H3K27me3 in both cell types. The prominent difference is that more promoters are marked by H3K9ac only or H3K9ac/H3K27me3 in hNECs than hESCs whereas promoters marked by H3K4me3/H3K9ac/H3K27me3 are opposite (Supplementary Figure S3b). This implies that H3K9ac likely has a critical role in the differentiation of hNECs. Analysis of HM dynamics shows that the majority of promoters remain their original chromatin state in the differentiation of hNECs from hESCs (Figure 3a and Supplementary Figure S3c). Moreover, the increased promoters marked by H3K9ac only in hNECs are from promoters marked by H4K3me3/H3K9ac in hESCs. The increased promoters marked by H3K9ac/H3K27me3 in hNECs are from promoters marked by H4K3me3/H3K9ac/H3K27me3 in hESCs. As a result, gene expression levels increase as active HM signals increase and/or repressive HM signals decrease, vice versa (Figure 3b and Supplementary Figure S3d).

We next specifically examined HM dynamics in the promoters with NDR formation during the differentiation. The active HM signals largely increase around TSS from hESCs to hNECs, especially H3K9ac levels are increased to approximately twofolds. Conversely, the repressive H3K27me3 is decreased (Figure 3c). There is also enrichment of RNA Pol II around the TSS and of H3K36me3 across the gene body that indicates active transcription of these genes (Figure 3d). This suggests that nucleosome eviction together with HM dynamics set up an open chromatin structure in the promoter regions in neuroectodermal genes for transcription to facilitate the differentiation of hNECs from hESCs.

### H3K9ac and H3K27me3 dynamics accurately regulates gene activity of distinct lineages

**Figure 4:**
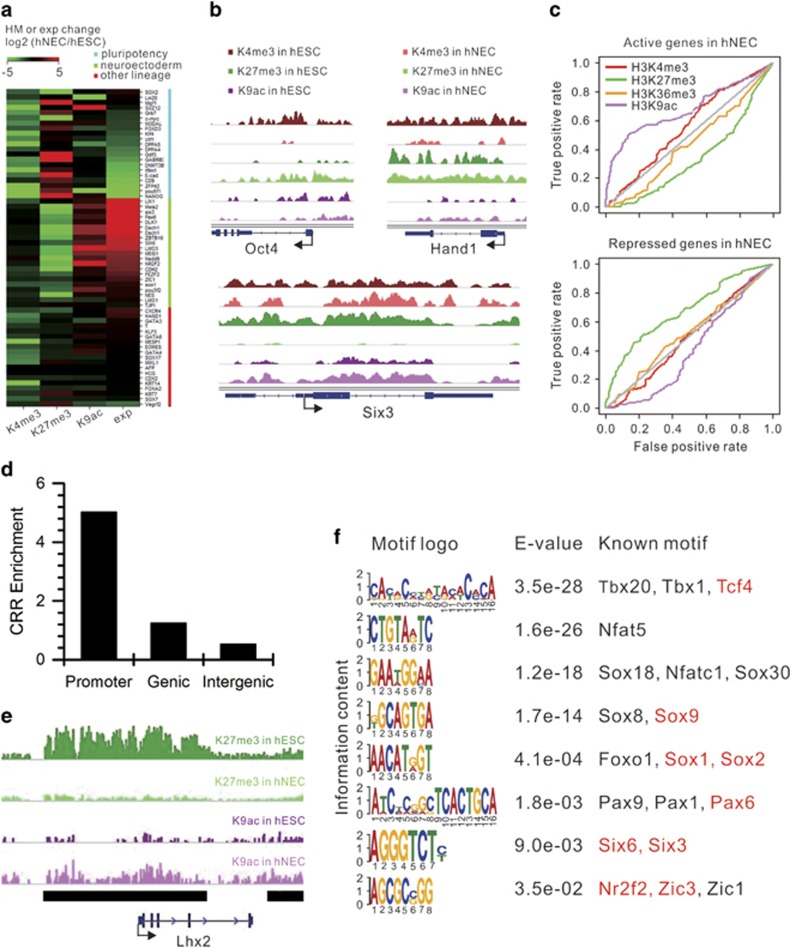
H3K9ac and H3K27me3 dynamics accurately regulates gene activity. (**a**) Changes of core histone modification signals in the promoters of three categories of genes ordered by expression changes within each category. (**b**) Track view of core histone modification signals in the representative genes of each category (Oct4: pluripotency, Hand1: other lineage, Six3: neuroectoderm). (**c**) Receiver operating characteristic (ROC) curves of four histone modifications as predictors of gene activity in hNECs. (**d**) Enrichment of chromatin remodeling regions (CRRs) in different genomic regions. The enrichment of CRRs in promoters is equal to the number of CRRs in promoters normalized by the length of promoters. The enrichment of CRRs in gene body and intergenic regions are calculated in the same way. (**e**) Track view of a representative CRR (labeled by the black line) in the promoter of neuroectodermal TF Lhx2. (**f**) Sequence motifs found in CRRs through *de novo* enrichment analysis. Corresponding E-values are indicated for each motif. The last column lists the TFs associated with similar motifs. Neuroectodermal TFs are in red

To address which HM(s) have a dominant role in control of gene activity in the differentiation of hNECs, we collected three sets of genes: pluripotency genes for hESCs, neuroectodermal genes for hNECs, and other lineage-specific genes for comparison, and examines HM dynamics in promoters and gene expression change. The results show that H3K4me3 levels largely decrease in pluripotency genes whereas H3K27me3 levels increase. H3K9ac levels change very little. Concomitantly, the gene expressions are downregulated (Figures 4a and b). This suggests that H3K27me3 and H3K4me3 have a pivotal role in regulation of pluripotency genes. Contrarily, H3K27me3 signals greatly decrease in neuroectodermal genes, whereas H3K9ac signals increase. H3K4me3 signals remain unchanged. Consequently, the gene expressions are upregulated (Figures 4a and b). Moreover, H3K9ac and H3K27me3 levels are the best predictive for gene activity in hNECs among the core HMs (Figure 4c). This indicates that H3K27me3 and H3K9ac have a dominant role in regulation of neuroectodermal genes. In contrast,there is very little change in both H3K27me3 and H3K9ac levels in other lineage-specific genes in concordance with unchanged gene expression levels (Figures 4a and b). This implies that other lineage-specific genes remain unchanged expression levels under the context of unchanged chromatin structure in promoters. ChIP-qPCR results confirmed the aforementioned distinct HM dynamics in promoters of the representative genes (Supplementary Figure S4a).

We further quantified change in H3K9ac and H3K27me3 signals in promoters upon differentiation by calculating the change in levels of the two HMs: log_2_(fold change of H3K9ac_hNECs/hESCs_)−log_2_(fold change of H3K27me3_hNECs/hESCs_) that is defined as Histone Modification Index (HMI). The distribution of HMI in genes is a normal distribution (Supplementary Figure S4b). The net effect of HMI⩽−1 is that increase in H3K9ac in promoters of these genes is equal to or less than two folds of increase in H3K27me3. Consistently, the expression levels of genes with HMI⩽−1 significantly decrease in hNECs. Conversely, the net effect of HMI⩾1 is that increase in H3K9ac in promoters of these genes is equal to or more than two folds of increase in H3K27me3. The expression levels of genes with HMI⩾1 significantly increase in hNECs. In contrast, there is no significant change in the expression levels of the rest of genes (Supplementary Figure S4c). This suggests that the combinational H3K9ac and H3K27me3 marks serve an appropriate predictive of global gene activity and have a critical role in the differentiation of hNECs from hESCs.

As increase in H3K9ac and decrease in H3K27me3 in promoters have a dominant role in activation of neuroectodermal genes (Figure 4a), we extended the similar analysis to the whole genome by identifying all regions prominent increase in H3K9ac or decrease in H3K27me3 upon differentiation, which are defined as chromatin remodeling regions (CRRs, see METHODS for details). There are total 7389 CRRs most of which have a length of 2000–6000 bp (Supplementary Figure S4d). Interestingly, CRRs are enriched in promoters (Figures 4d and e; Supplementary Figure S4e). Moreover, CRRs contain binding motifs of many TFs associated with neural differentiation and development, including Pax6, Six6, and Sox family (Figure 4f). Collectively, H3K9ac and H3K27me3 execute their epigenetic regulatory functions during the differentiation through chromatin remodeling mainly in promoters and consequently likely controlling accessibility of binding motifs of key TFs.

### Sox2 binds to hNEC-specific sites upon the differentiation

**Figure 5:**
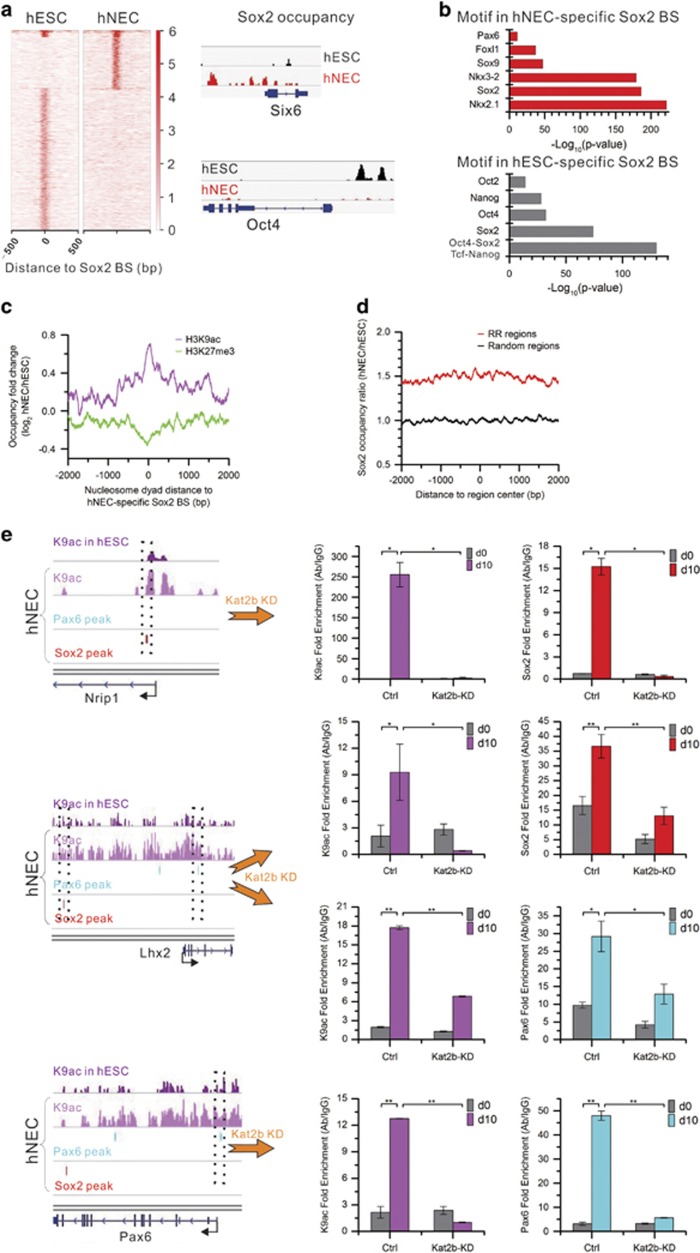
Kat2b acetylates the target sites of Sox2 and Pax6 for their binding. (**a**) Sox2 binds to distinct sites in hESCs and hNECs (heat map). Track view of Sox2-binding signals in the promoters of neuroectodermal TF Six6 and pluripotency TF Oct4. (**b**) Enrichment of co-factor motifs in hESC- and hNEC-specific Sox2-binding peak sequences. (**c**) Fold change of core histone modification signals in the vicinity of hNEC-specific Sox binding sites. (**d**) Fold change of Sox2 occupancy in ±2 kb regions of CRRs' center. (**e**) Kat2b knockdown reduces H3K9ac levels and the binding of Sox2 and Pax6. Left: track view of H3K9ac signals in the regions spanning Sox2 or Pax6 binding peaks in the enhancer or promoter of three neuroectodermal TFs (Nrip1, Lhx2, Pax6) in the wild type cells. Right: qPCR validation of significantly decreased H3K9ac signals and the occupancy of Sox2 and Pax6 at their binding sites indicated by the dotted boxes in the left panel in hNECs after Kat2b knockdown. Error bars represent SEM. (*P<0.05, **P<0.01, Student's *t*-test)

TF Sox2 has important roles in both ESCs and progenitors of multiple lineages.^22, 23^ How Sox2 executes its distinct functions, especially in the differentiation of hNECs, remains largely unknown. Our highly reproducible Sox2 ChIP-seq data (Supplementary Figure S5a) identified 12,623 binding sites in hESCs and 4875 sites in hNECs. Most are unique in each cell type except 411 sites common in both cell types (Figure 5a). The binding site sequences are abundant with Sox2-binding motif that resembles the canonical Sox2 motif in the Cistrome database^27^ (Supplementary Figure S5b). This suggests that our Sox2 ChIP-seq data capture its bona fide target sites. *De novo* motif discovery further identified motifs of other pluripotency TFs, including Oct4, Nanog, and so on, in hESC-specific Sox2-binding sites. In contrast, motifs of other neural TFs, including Nkx2.1, Sox9, Pax6, and so on, are enriched in hNEC-specific Sox2-binding sites (Figure 5b). This together indicates that Sox2 exerts its distinct functions in pluripotency maintenance and multilineage progenitor commitment by binding to distinct target sites with involvement of other corresponding co-factors.

### Kat2b deposits H3K9ac to recruit Sox2 to its target sites

To understand how chromatin remodeling regulates Sox2 binding to its target sites upon differentiation, we examined nucleosome occupancy around hNEC-specific Sox2-binding sites and found no change during the differentiation (Supplementary Figure S5c). However, H3K9ac levels are largely increased on the sites whereas H3K27me3 levels are decreased (Figure 5c). Moreover, Sox2-binding signals are enriched in CRRs (Figure 5d). This implies that H3K9ac and H3K27me3 are a dominant factor for recruitment of Sox2 to the target sites other than nucleosome occupancy.

Acetyltransferase Kat2b is the key enzyme responsible for H3K9ac deposition. Coincidently, Kat2b is significantly upregulated upon differentiation. Thus, we hypothesized that Kat2b may be the key factor to regulate Sox2 binding by depositing H3K9ac upon differentiation. To test this, we knocked down Kat2b by shRNAs (Supplementary Figure S5d). As knockdown of Kat2b, H3K9ac levels are significantly decreased at Sox2-binding sites, and Sox2 occupancy is also significantly reduced. Similarly, Kat2b knockdown also significantly reduces H3K9ac levels at Pax6 target sites and consequently Pax6 binding signals are precipitously decreased (Figure 5e). As a result, Kat2b knockdown fails the differentiation of hNECs from hESCs (Supplementary Figure S5e).

## Discussion

**Figure 6:**
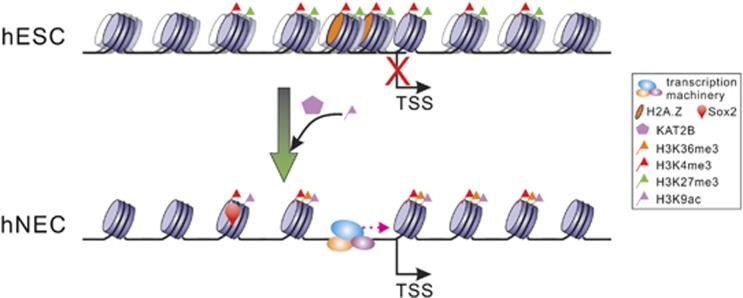
Model of how Kat2b epigenetically activates neuroectodermal genes upon differentiation. In hESCs, nucleosome positioning is overall fuzzy. Nucleosomes are densely packed in promoters that are at a repressive chromatin state with prevalent H3K27me3 marks. Upon differentiation, nucleosome positioning becomes phased. H3K27me3 signals are greatly reduced. Concomitantly, Kat2b deposits H3K9ac signals that enhance Sox2 binding. H2A.Z-mediated nucleosomes eviction creates an open chromatin structure for transcription machinery assembly. As a result, neuroectodermal genes are activated for neuroectodermal commitment

Chromatin remodeling has a critical role in cell differentiation. In this study, the results show there occur extensive nucleosome repositioning and HM dynamics during the differentiation of hNECs from hESCs. H2A.Z mediates nucleosome eviction to form NDRs in the promoters of neuroectodermal genes. Acetyltransferase Kat2b deposits H3K9ac in the genes in concomitance with decrease in repressive H3K27me3 levels. The resultant open chromatin structure in the promoters activates neuroectodermal gene transcription. Meanwhile, the increased H3K9ac signals help recruit Sox2 to its target sites unique to hNECs. As a result, hESCs are directly differentiated to hNECs (Figure 6). These findings reveal the dynamics of chromatin structure and its epigenetic regulatory roles in the differentiation of hNECs from hESCs. This indicates that the fine-tuning of chromatin structure is critical to lineage-commitment gene regulation.

Dynamic changes in chromatin modifications have crucial roles in cell differentiation. A previous study illustrated that H3K4me3 at promoters remains largely invariant during hESCs differentiating to a mesendodermal lineage. In contrast, switch between H3K27ac and H3K27me3 at promoters is critical to the differentiation by repressing pluripotency genes and activating mesendodermal genes.^28^ Intriguingly, our findings suggest that dynamic changes in H3K9ac and H3K27me3 at promoters are important to the differentiation of hNECs from hESCs. Consistently, H3K9ac levels at promoters are critical to glial and neuronal commitment in *Drosophila* embryonic neural development.^4, 5, 29^ Collectively, marked changes in distinct combinational histone modifications in cell fate commitment tend to be cell-type specific.

Nucleosome occupancy is fundamental for chromatin structure. Nucleosome eviction leads to an open chromatin structure increasing accessibility of DNA sequences and therefore has an important role in the differentiation process. It was reported that chromatin remodeling complexes SWI/SNF and INO80 participate nucleosome depletion in promoters during differentiation toward the endoderm/hepatic fate.^3^ Similarly, we observed H2A.Z-mediated nucleosome eviction in promoters upon differentiation of hNECs than significantly increase transcription levels of neuroectodermal genes. However, the detailed mechanism of which chromatin remodeling complexes orchestrate to regulate nucleosome eviction requires future studies.

Interplay between TFs and histone post-translational modifications precisely regulates transcription. Chromatin modifications enhance recruitment of TFs to certain genomic regions. For example, TFs and co-activators favor binding to transcriptional enhancers defined by H3K4me1 enrichment. Extended H3K27ac domains are abundant of motifs for master TFs in the respective cell types.^30, 31^ Here, we reveal that H3K9ac marks at the hNEC-specific binding sites of neuroectodermal factors Sox2 and Pax6 are essential for their binding. Knockdown of acetyltransferase Kat2b largely reduces H3K9ac levels, disrupts bindings of Sox2 and Pax6, and consequently fails neuroectodermal differentiation. This greatly improves our understanding of regulatory roles of chromatin remodeling in the differentiation of hNECs from hESCs.

## Materials and methods

### Differentiation of hNECs from hESCs

Human ESCs (H9, passages 18–21) were differentiated into hNECs as described previously.^19, 21^ Briefly, hESC colonies were cultured on irradiated mouse embryonic fibroblasts (MEFs) in ESC growth medium (Sterilely combine 392.5 ml DMEM/F12, 100 ml Knockout serum replacer, 5 ml MEM nonessential amino acids solution, 2.5 ml of 200 mM L-glutamine solution (final concentration of 1 mM), and 3.5 ml 14.3 M b-Mercaptoethanol (final concentration of 0.1 mM)) supplemented with fresh 4 ng/ml recombinant human FGF basic (bFGF, Invitrogen, Waltham, MA, USA, Cat. No. 13256-029). Then, the colonies were detached from MEFs and formed aggregates to initiate the neural differentiation procedure by withdrawing bFGF. After 4 days of suspension culture, the ESC growth medium was replaced with neural induction medium (Sterilely combine 489 ml of DMEM/F12, 5 ml N2 supplement, 5 ml MEM nonessential amino acids solution, and 1 ml of 1 mg/ml Heparin) to guide neuroectodermal specification. Then transferred the suspension cells to the 6-well plates without feeder on the sixth day. After another 4 days of adherent growth, we got hNECs on day 10.

### Immunofluorescence staining

Cells were fixed by 4% paraformaldehyde at room temperature for 10 min, followed by 0.1% Triton X-100 permeabilization for 10 min and 1% BSA blocking for 1 h. They were then incubated with primary antibodies against Oct4 (1:500, Santa Cruz, Dallas, TX, USA), Nanog (1:500, R&D, Minneapolis, MN, USA), Sox2 (1:500, R&D) or Pax6 (1:500, DSHB, Iowa City, IA, USA) at 4 °C overnight. The next day, cells were incubated with secondary antibodies against mouse or goat IgG (Life Technologies, Waltham, MA, USA) accordingly. Nuclei were counterstained with Hoechst (Invitrogen), and confocal images were taken on the Zeiss LSM 710 microscope (Goettingen, Germany).

### mRNA extraction and qRT-PCR

Total RNA was isolated using the Trizol reagent (Invitrogen) and RNA concentration was determined by NanoDrop 2000 c (Thermo Scientific, Waltham, MA, USA). Overall, 1 *μ*g of total RNA was reversely transcribed into cDNA using SuperScript III (Invitrogen) and subjected to real-time PCR (Bio-Rad, Hercules, CA, USA, CFX Connect Real-Time System) using the Ssofast EvaGreen kit (Bio-Rad). The real-time PCR primers were listed in the Supplementary Table S1.

### MNase-seq and ChIP-seq for histone modifications

Overall, ~2 × 10^7^ cells were crosslinked with 1% formaldehyde for 10 min at room temperature, and crosslinking was terminated by addition of 125 mM glycine. Cells were harvested for lysis to isolate nuclei. Suspend nuclei in 500 *μ*l of MNase digestion buffer (10 mM Tris-HCl (pH 7.5), 15 mM NaCl, 60 mM KCl, 1 mM CaCl_2_, 0.15 mM spermine, 0.5 mM spermidine, 1 × EDTA free protease inhibitor cocktail). Then digest nuclei with 2.5 *μ*l of micrococcal nuclease (NEB# M0247S, 2000 gel units/*μ*l) at 37 °C for 20 min. Then put the samples on ice and add EDTA to a final concentration of 10 mM to stop MNase digestion. Crosslinking was then reversed for at least 6 h at 65 °C along with proteinase K digestion. Nucleosomal DNA was extracted using phenol–chloroform and purified on 1.5% agarose gels.

A total of 10–20 *μ*g of nucleosomal DNA was used for chromatin immunoprecipitation with 3–5 *μ*g of HM antibodies as described in 400-*μ*l ChIP buffer (2 mM EDTA, 150 mM NaCl, 20 mM Tris-HCl (pH 8.1), 0.1% Triton X-100, 1XEDTA free protease inhibitor cocktail). Antibodies used are from Abcam (Cambridge, MA, USA): H3K4me3 (ab8580), H3K27me3 (ab6002), H3K36me3 (ab9050), H3K9ac (ab10812). The mixture was incubated overnight at 4 °C with rotation. Then, 30 *μ*l of ChIP-Grade Protein G Magnetic Beads (CST; #9006) were added in the mixture for another 2 h. After that, beads were sequentially washed three times with low salt buffer (2 mM EDTA, 20 mM Tris-HCl (pH 8.1), 0.1% SDS, 1% Triton X-100, 150 mM NaCl), once with high salt buffer (2 mM EDTA, 20 mM Tris-HCl (pH 8.1), 0.1% SDS, 1% Triton X-100, 500 mM NaCl), once with LiCl wash buffer (0.25 M LiCl, 1% NP-40, 1% sodium deoxycholate, 1 mM EDTA, 10 mM Tris-HCl (pH 8.1)), and once with TE buffer (10 mM Tris-HCl (pH 8.1), 1 mM EDTA). Then beads were suspended in elution buffer (50 mM Tris-HCl (pH 8.1), 10 mM EDTA, 0.1–0.5% SDS) at 65 °C for 1 h.

The purified mononucleosomal DNA by MNase digestion and ChIP'ed by HM antibodies were subjected to massively parallel DNA sequencing on Illumina HiSeq2000 platform (San Diego, CA, USA) using 49 bp single end protocol.

### ChIP-seq for Sox2 and RNA Pol II

Genomic DNA was crosslinked and extracted as described above MNase-seq. The purified genomic DNA was suspended in 500 *μ*l of sonication buffer (1XPBS, 1% NP-40, 0.5% sodium deoxycholate, 0.1% SDS, 1 × EDTA free protease inhibitor cocktail), and sonicated with 6 rounds of 30-s on and 30-s off on ice using XL-2000 Misonix sonicator with power output of 7 Watts. It is critical that the average length of the sheared chromatin is about 250 bp, with length ranging from 150–500 bp. The fragmented DNA was immunoprecipitated with 3~5 *μ*g of specified antibody (anti-Sox2: Abcam ab59776, anti-Pol II: Abcam ab817) as described in the above ChIP-seq for HMs. The ChIP'ed DNA was subjected to massively parallel DNA sequencing on Illumina HiSeq2000 platform (San Diego, CA, USA) using 49 bp single end protocol.

### ChIP-qPCR

ChIP-qPCR was conducted according to user manual of ChampionChIP PCR Array from SABiosciences (Germantown, MD, USA). Briefly, DNA fragments were prepared as above MNase-seq or ChIP-seq. Relative signal abundance in regions of interest in sample DNA was measured by qPCR using Power SYBR. Fragmented genomic DNA or IgG-immunoprecipitated DNA was used as control sample. Relative signal enrichment was calculated using ΔΔCt method by normalizing Ct values against control sample. The ChIP-qPCR primers were were listed in the Supplementary Table S2.

### Knockdown of Kat2b in hESCs

We knocked down Kat2b in hESCs by TALEN. CAG-GFP fragment was replaced by U6 promoter and shRNA sequence from AAV-CAGGS-EGFP (Addgene, Cambridge, MA, USA). The shRNA targeting sequences were: shKat2b#1 (5′-CGAACTCTAATCCTCACTCAT-3′), shKat2b#2 (5′-CCAGCCAGCTAGGCATCCAAA-3′), shKat2b#3 (5′-GGAAGCTGGATTAATTGACAA-3′). Before electroporation, hESCs were cultured in ROCK-inhibitor Y-27632 (Millipore, Hayward, CA, USA, 688002) for 3 h. About 1 × 10^7^ cells were mixed with 5 *μ*g of each TALEN targeting AAVS1 locus.^32^ And 40 *μ*g of AAVS1-SA-PURO-PA-U6 promoter-shRNA-PA donor after trypsin/EDTA solution treatment and then electroporated (Gene Pulser Xcell System, Bio-Rad: 250 V, 500 *μ* F, 0.4 cm cuvettes). Transfected cells were plated on MEF with ROCK-inhibitor for the first 24 h. Then we used 1 *μ*g/ml puromycin (Sigma, St. Louis, MO, USA, P8833) to select cells cultured with MEF-conditioned medium. Individual colonies were picked up after about 15 days and identified by PCR using primers for homologous recombination forward: 5′-CTTCCGCATTGGAGTCGCTTTA-3′ and reverse: 5′-ACAGGAGGTGGGGGTTAGAC-3′ or wild type forward: 5′-CAGCCGGTCCTGGACTTTGTC-3′ and reverse: 5′-AGCCGGGAACCGCTCAACTC-3′.

### Microarray analysis

Gene expression microarray data of the two cell types were downloaded from ArrayExpress database (accession number E-MEXP-2426, Affymetrix HG-U133A).^33^ The data contained 3 replicates. The.CEL files were analyzed using the affy package within R/Bioconductor.^34^ Robust multichip averaging (RMA) was used to correct for background, normalize and generate expression data.^35^ The Limma (linear models for microarray data) package was then used to identify differentially expressed genes with the adjusted *P*-value<0.05 and expression fold change ⩾2.^36^

### Functional annotation of gene sets

GO analysis for gene sets was performed using the tool PANTHER.^37^ The tool Gene Set Enrichment Analysis (GSEA v2.1.0)^24^ was used to identify any priori defined set of genes that share common biological function or regulation and show statistically significantly, concordantly different expression levels between two samples. GSEA was performed using the gene sets of gene ontology (C5) and user-defined HESC SPECIFIC MARKERS gene set that was collected from published paper.^38^ Metric for ranking genes was set to Signal2Noise. Gene ontology gene sets were curated by MSigDB database v4.0. Other parameters used default values.

### Nucleosome prediction and analysis of nucleosome positioning dynamics

Sequencing reads were aligned to *Homo sapiens* reference genome (hg19) using Bowtie-1.0.0 (ref. 39) with up to two mismatches. Only the uniquely mapped reads were retained for nucleosome prediction. Because only the border of mononucleosomal DNA was sequenced, each read was moved 73 bp interior to its end to represent nucleosome dyad. The peak-calling tool GeneTrack^25^ was used to smooth the clustered distribution of reads and predict nucleosomes using an exclusion zone of 147 bp and sigma of 20 bp. Nucleosomes that were detected six or more times (that is, read count ⩾6) were further analyzed, although patterns were identical when all nucleosomes were analyzed. Nucleosome fuzziness was calculated as the standard deviation of the coordinates of all reads defining the same nucleosome as described previously.^40^ It measures how spread out a nucleosome position is. Each nucleosome was assigned to promoter, genic or intergenic regions depending on the location of the nucleosome midpoint.

Two nucleosomes from the two cell types are fixed if their midpoints locate at the same position. The nucleosome is gain or loss if the distance between two midpoints is ⩾107 bp. That is, the two nucleosomes overlap less than 20 bp. The rest of nucleosomes shift upon differentiation.

### Nucleosome distribution profile around TSS

The original composite distribution of nucleosome around TSS was calculated by aggregating nucleosomal read count at each distance relative to TSS as follows: each read represents a nucleosome by extending toward 3′ end to a length of 147 bp. The midpoint of extended read defines the nucleosome position. We summed total read counts at each site within ±2 kb of TSS. The nucleosomal read count was further normalized by the uniquely mapped total reads. We further binned the nucleosome occupancy by a 10- bp interval of nucleosome distance to TSS, and smoothed it with 5-bin moving average and 1-bin step size.

Nucleosome organization in the regions −0.3 kb to +1 kb of TSS was plotted as heat map by the tool seqMINER^41^ without reads extension. Nucleosome positions predicted by GeneTrack was used as input with nucleosome width shrunk to 127 bp for better visualization. Heat map for nucleosomes with H3K36me3 modifications was drawn in the same manner. Heat map for RNA Pol II was drawn in the similar way except that its peaks predicted by MACS-1.4.2^(ref. 42)^ with default settings were used.

### Chromatin state in promoters

HM ChIP-seq reads were aligned to *Homo sapiens* reference genome (hg19) as above nucleosomal reads. Similarly, only the uniquely mapped reads were retained for prediction of nucleosome with HM by GeneTrack using the same parameters as above nucleosome prediction. Occupancy of nucleosomes with HM were normalized as Reads Per Kilobase per Million mapped reads (RPKM). Nucleosomes with HM that had occupancy as RPKM>1 were retained for following analysis. Promoters (±500 bp of TSS) were categorized to eight classes (H3K4me3+, H3K27me3+, H3K9ac+, H3K4me3+/H3K27me3+, H3K4me3+/H3K9ac+, H3K9ac+/H3K27me3+, H3K4me3+/H3K27me3+/H3K9ac+, none) depending on the composition of nucleosomes with modifications located in promoters. For example, H3K4me3+ promoters contained only nucleosomes with H3K4me3, H3K4me3+/H3K27me3+ contained only both nucleosomes with H3K4me3 and nucleosomes with H3K27me3, and so on.

### Change in HMs in promoters

Occupancy of a HM was measured as its read counts in promoters that were normalized as reads per kilobase per million mapped reads (RPKM). HM occupancy ratio of hNECs to hESCs was calculated, transformed to log2, and represented as heat map.

We further used ROC curve to identify which HM(s) were the best classifier for the significantly DEGs. In first case, upregulated DEGs were treated as actual positives, the rest were actual negatives. The fold changes of HM occupancy in promoters were calculated as above and sorted descendingly. Each fold change value was used as a threshold. For each threshold, the genes, whose fold changes of HM occupancy in promoters were greater than the threshold, were predictive positives. Otherwise, the genes are predictive negatives. Then, the true positive rate (TPR, that is, sensitivity) and false positive rate (FPR, that is, 1–specificity) were calculated for each threshold. The ROC curve was thus TPR as a function of FPR. The ROC curve for downregulated DEGs was plotted in the same manner except that downregulated DEGs were treated as actual positives, the rest were actual negatives.

### Chromatin remodeling regions

The regions with significant increase in H3K9ac upon differentiation were detected by the tool MACS-1.4.2^(ref. 42)^ with a *P*-value cutoff of 10^−5^ using hNECs as treatment sample and hESCs as background. The regions with significant decrease in H3K27me3 upon differentiation were detected similarly using as hESCs treatment sample and hNECs as background. We retained the regions with H3K9ac or H3K27me3 occupancy >1 RPKM. We combined these regions as chromatin remodeling regions. *De novo* motif discovery in these regions was done by the tool MEME^43^ as follows: the DNA sequences of CRRs were retrieved as input to MEME for motif finding with E-value cutoff of 0.05 and motif width of 6–16 bp. The other parameters used default values. To compare the predicted motifs to the known ones, the TOMTOM motif comparison tool (Version 4.11.3) from the MEME suite was used to search a database of vertebrates (*in vivo* and in silico) using the default parameters (*E*-value<10).

### Profiles of Sox2 binding and motif finding in Sox2 peaks

As above HM sequencing data analysis, only Sox2 reads uniquely mapped to *Homo sapiens* reference genome were retained for peak calling by the tool MACS-1.4.2 (ref. 42) with a *P*-value cutoff of 10^−4^ and fold change of 10. The *de novo* motif finding in the Sox2 peak sequences was done by Cistrome Analysis Pipeline^27^ as follows: we chose the SeqPos motif tool in the section Integrative Analysis from the Cistrome toolbox. The coordinates of Sox2 peaks in BED format was the input file. The ‘*de novo* motif search' motif database from the ‘curated cistrome motif database' was used. The species ‘*Homo sapien* or *Mus musculu*s' was used. The rest of parameters were default. The tool HOMER was applied to find enriched motifs of co-factors.^44^ Only the motifs with the highest alignments to known transcription factors, nonredundant matrixes and non-repetitive sequences were retained. Sox2 occupancy in peaks was normalized as RPKM. Browser tracks for Sox2 occupancy were generated using the tool IGV 2.3.^45^

### Accession numbers

The accession numbers for the MNase-seq data and the ChIP-seq data reported in this paper are GEO: GSE76084. The H2A.Z ChIP-seq data was downloaded from GSM1003579. Pax6 binding peaks in hNECs was kindly provided by the author from the published work.^46^
