# Supplementary material for: Nucleosome eviction along with H3K9ac deposition enhances Sox2 binding during human neuroectodermal commitment
Source: Cell Death Differ. 2017 May 5;24(6):1121–31. doi: 10.1038/cdd.2017.62 (PMC5442478; doi:10.1038/cdd.2017.62)
Supplement: Supplementary Material [file cdd201762x1.pdf]

**Supplemental Material**

# Nucleosome eviction along with H3K9ac deposition enhances Sox2 binding during human neuroectodermal commitment

Yanhua Du[1,2], Zhenping Liu[2], Xinkai Cao[2], Xiaolong Chen[2], Zhenyu Chen[3], Xiaobai Zhang[2], Xiaoqing Zhang[1,3,*], Cizhong Jiang[1,2,*]

[1] Institute of Translational Research, Tongji Hospital, Tongji University, Shanghai 200065, China

[2] The School of Life Sciences and Technology, Shanghai Key Laboratory of Signaling and Disease Research, the Collaborative Innovation Center for Brain Science, Tongji University, Shanghai 200092, China

[3] Tongji University School of Medicine, Shanghai 200092, China

* **Correspondence:**

**Supplemental figures and legends**

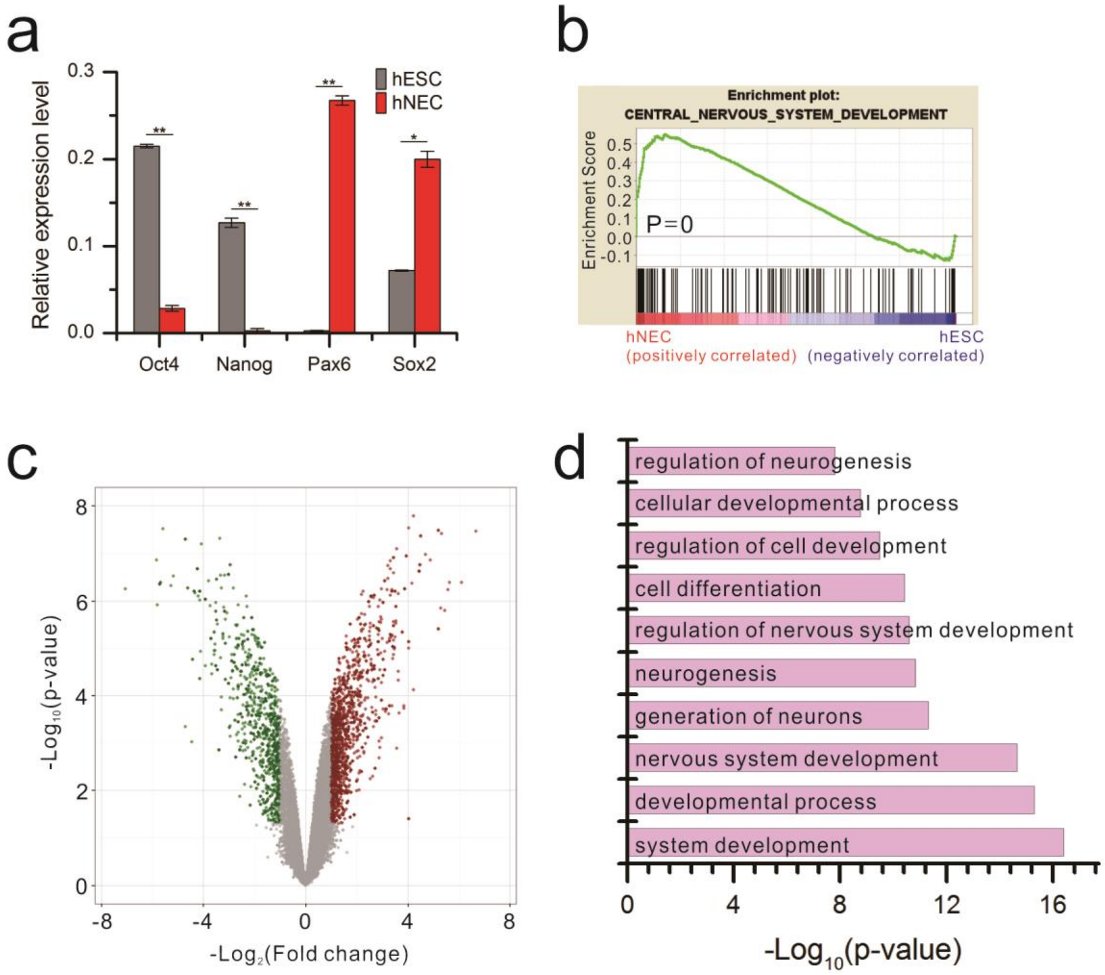


**Supplementary Figure S1.** Expression profiles along hESCs differentiating to hNECs. (**a**) qPCR results of representative marker genes. Error bars are SEM. (* $p < 0.05$, ** $p < 0.01$, Student's T-test). (**b**) GSEA results: "central nervous system development" genes are significantly up-regulated in hNECs. (**c**) Volcano plot showing gene expression profiles upon differentiation. Significantly differentially expressed genes are in colors (fold change ≥ 2 and p-value < 0.05; red: up-regulated, green: down-regulated). (**d**) Enriched GO terms in significantly up-regulated genes in hNECs.

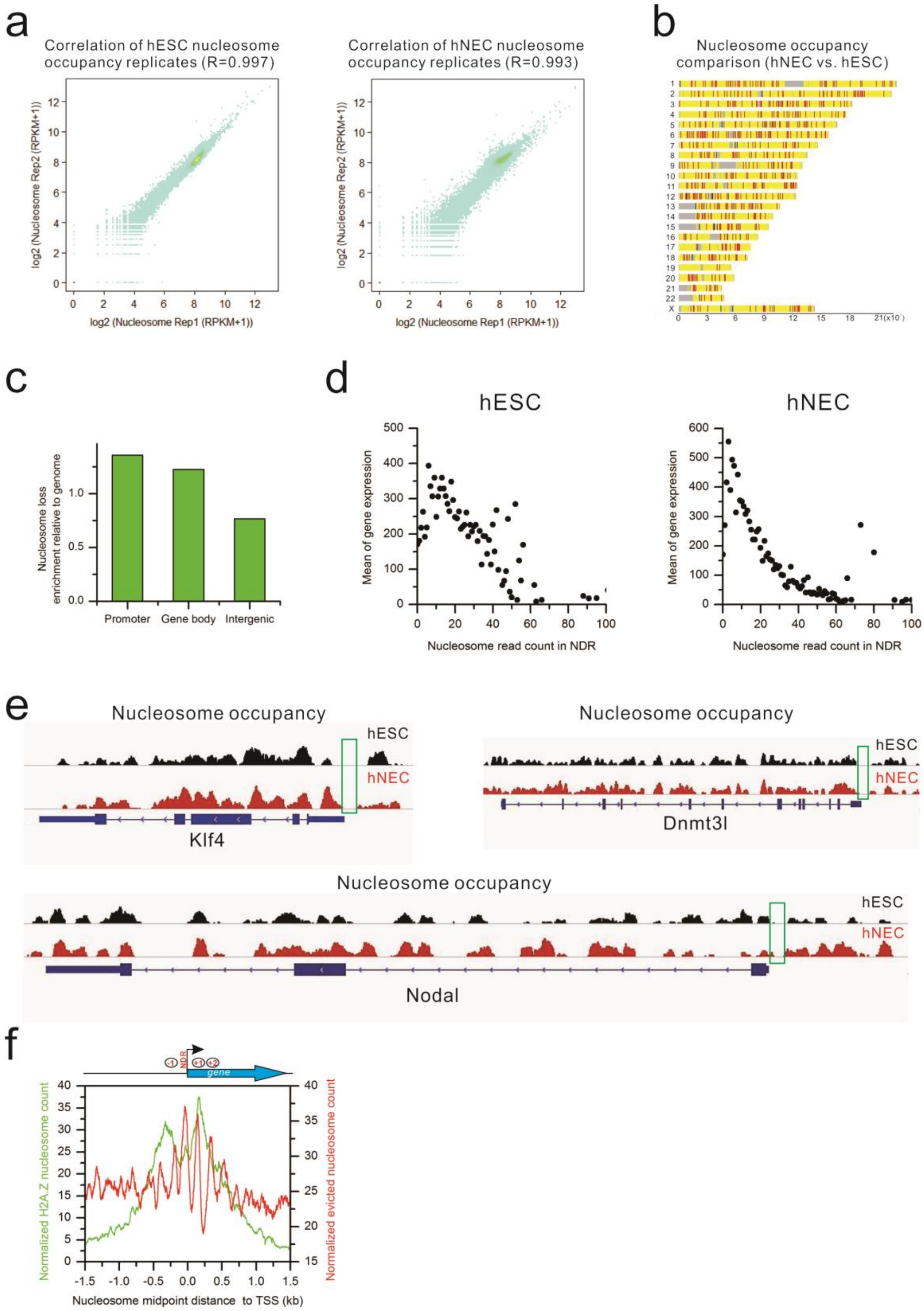


**Supplementary Figure S2.** Nucleosome positioning dynamics upon differentiation.
(**a**) We scanned the genome with a 10 kb window and calculated the normalized nucleosome occupancy for each window. The high correlation of the global nucleosome occupancy between the biological replicates indicates the high reproducibility (Pearson correlation). (**b**) There is significant difference in nucleosome occupancy upon differentiation. The nucleosome occupancy is calculated as in (**a**). The ratio of normalized nucleosome occupancy

within each window is presented by a color. Red indicates nucleosome occupancy in hNECs is at least 1.5 fold of that in hESCs. Green indicates nucleosome occupancy in hESCs is at least 1.5 fold of that in hNECs. Yellow indicates the ratio is less than 1.5 fold. Grey indicates regions of Ns in the genome. (**c**) Enrichment of evicted nucleosomes in different genomic regions. The enrichment of nucleosome loss in promoters is equal to the number of evicted nucleosomes in promoters normalized by the length of promoters. The enrichment of nucleosome loss in gene body and intergenic regions are calculated in the same way. (**d**) Black dotted curve showing negative correlation between nucleosome occupancy in promoter NDRs and gene expression level. Pearson's correlation coefficients are -0.71 for hESCs and -0.89 for hNECs. (**e**) Track view of maintained NDR in the promoter of three pluripotency TFs during differentiation. Green boxes indicate the NDRs. (**f**) Distribution of H2A.Z and evicted nucleosomes around TSS. Notably, due to the low resolution of H2A.Z nucleosomes (from GSM1003579), it does not allow us to see the mononucleosomes around TSS. The canonical -1, NDR (Nucleosome Depletion Region), +1, +2 nucleosome arrangement around TSS is indicated on the top.

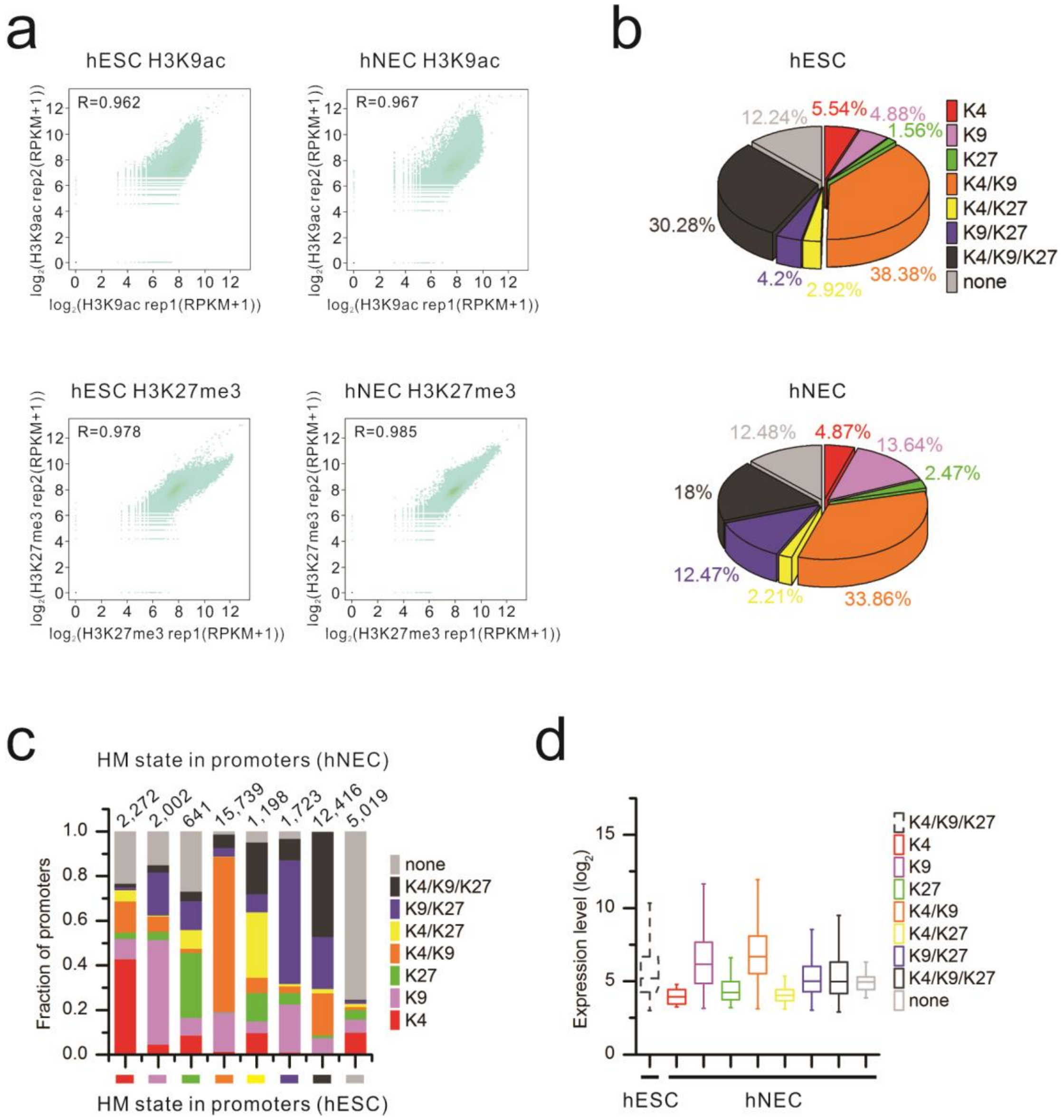


**Supplementary Figure S3.** Histone modification changes in the promoter regions.
(**a**) We scanned the genome with a 10 kb window and calculated histone modification occupancy for each window. The high correlation of the global histone modification signals between the biological replicates indicates the high reproducibility (Pearson correlation). (**b**) Distribution of each type of promoters categorized by histone modification content. (**c**) Statistics summary of each category of promoters defined by histone modification mark(s) in hESCs changing into other categories in hNECs. The number of promoters for each category are indicated above bars. (**d**) Changes in expression levels of genes with H3K4me3 / H3K9ac / H3K27me3 in hESCs upon differentiation to hNECs. Loss of active H3K9ac is associated with decreased expression. Conversely, loss of repressive H3K27me3 is associated with increased expression.

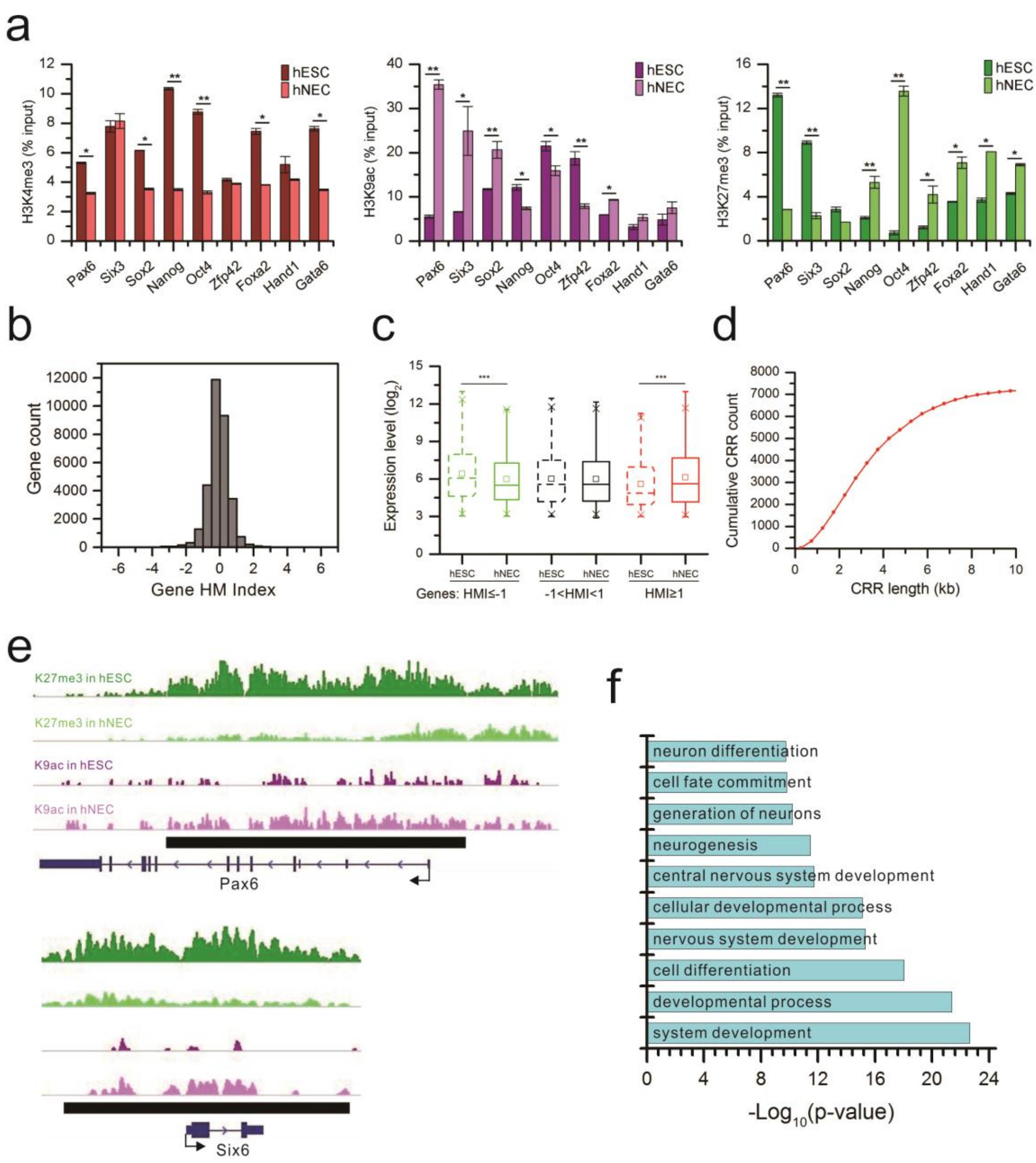


**Supplementary Figure S4.** Chromatin state dynamics along differentiation.
(**a**) ChIP qPCR results of core histone modification occupancy in the promoters of representative neuroectodermal (Pax6, Six6), pluripotency (Sox2, Nanog, Pou5f1, Zfp42), and other-lineage (Foxa2, Hand1, Gata6) genes. Error bars are SEM. (* $p < 0.05$, ** $p < 0.01$, Student's T-test). (**b**) Histogram showing the distribution of gene histone modification index (HMI). HMI = $\log_2(\text{fold change}_{H3K9ac}) - \log_2(\text{fold change}_{H3K27me3})$ in the promoter regions (±500 bp of TSS). (**c**) Expression profiles of genes classified by HMI. (*** $p < 0.001$, Student's T-test). (**d**) Cumulative frequency of CRR length. (**e**) Track view of two representative CRRs (labeled by the black line) in the promoters of neuroectodermal TFs Pax6 and Six6. (**f**) GO terms enriched in the genes associated with CRRs.

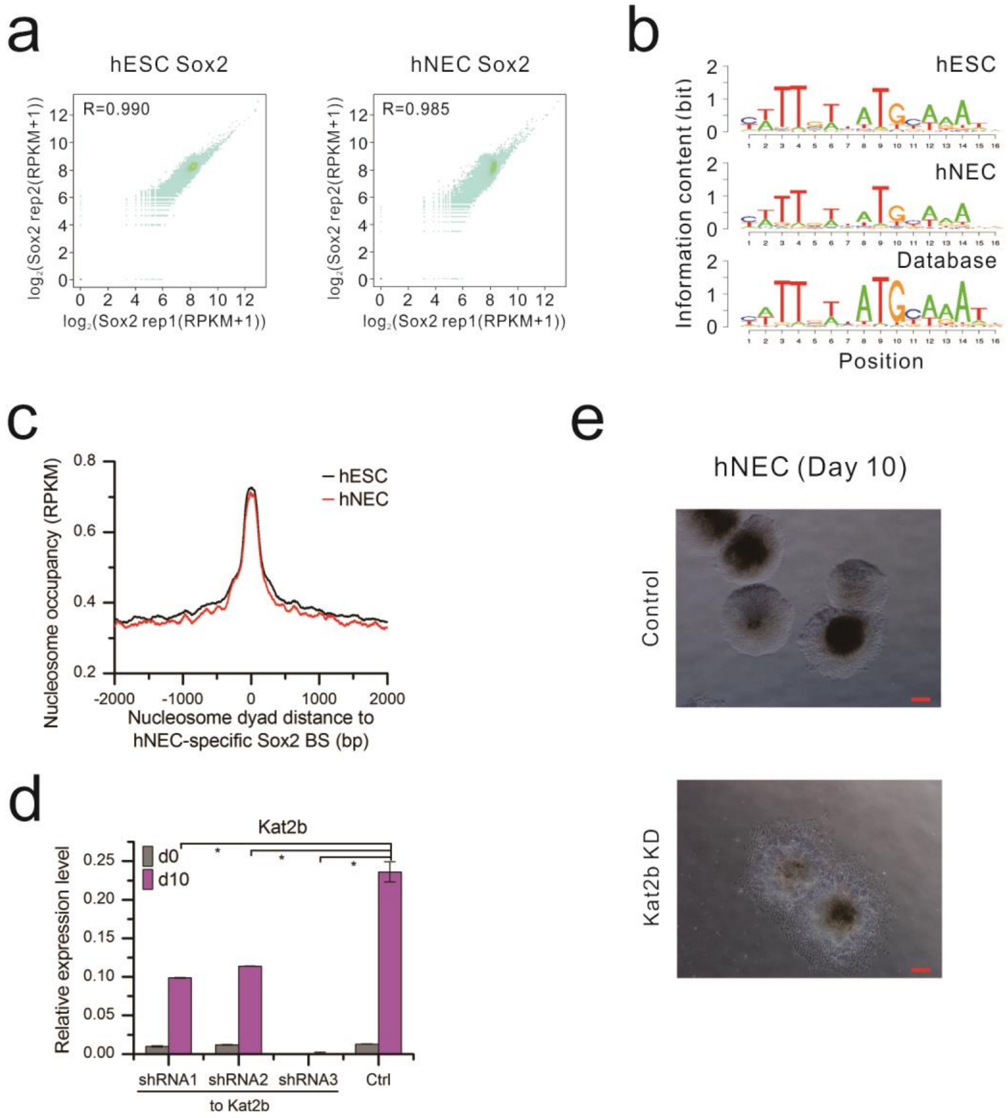


**Supplementary Figure S5.** Impact of Kat2b knockdown on neuroectodermal commitment. (**a**) We scanned the genome with a 10 kb window and calculated Sox2 occupancy for each window. The high correlation of the global Sox2 occupancy between the biological replicates indicates the high reproducibility (Pearson correlation). (**b**) *De novo* sequence motifs overrepresented in hESC- and hNEC-specific Sox binding sites, and the canonical Sox2 motif from Cistrome database. (**c**) Nucleosome occupancy around hNEC-specific Sox2 binding sites during the differentiation. (**d**) qPCR validation of significantly decreased Kat2b expression in hNECs after Kat2b knockdown. Error bars represent SEM. (* $p < 0.05$, Student's T-test). (**e**) Changed cell morphology by Kat2b knockdown that fails hESCs differentiating to hNECs. Scale bars, 100 µm.

**Supplementary Tables**

**Supplementary Table S1.** qRT-PCR primers for examination of expression levels of selected genes.

| Gene | Forward (5'-3') | Reverse (5'-3') |
|---|---|---|
| Pax6 | TCTTTGCTTGGGAAATCCG | CTGCCCGTTCAACATCCTTAG |
| Oct4 | ACATCAAAGCTCTGCAGAAAGAACT | CTGAATACCTTCCCAAATAGAACCC |
| Nanog | ATTCTTCCACCAGTCCCAAA | ATCTGCTGGAGGCTGAGGTA |
| Sox2 | GCCCTGCAGTACAACTCCAT | TGGAGTGGGAGGAAGAGGTA |
| Kat2b | CTGGAGGCACCATCTCAACGAA | ACAGTGAAGACCGAGCGAAGCA |
| Gapdh | GAAGGTGAAGGTCGGAGTC | GAAGATGGTGATGGGATTTC |

**Supplementary Table S2.** ChIP-qPCR primers for examination of nucleosome occupancy in promoter NDRs of selected genes.

| Gene | Forward (5'-3') | Reverse (5'-3') |
|---|---|---|
| Pax6-1 | GGGCAGCCAATGAGGACG | TTACCTGGGAGCGGAGGG |
| Pax6-2 | GCCATTTACTTACTTTCCTCC | GTGTCCCACTGGCGATGT |
| Pax6-3 | TTCATATTCAAACAAACGGACC | TTCCCTTTTCAAACCCACTAA |
| Meis2-1 | TTTCCTCCTGTGCTTCCC | AGTCATCTGGGTCCGATGTAG |
| Meis2-2 | TAAGAAAGTGATCTAGGCTGAA | GAGGAGGAAGAAGAGGAAAA |
| Meis2-3 | AGAGGGAGGGAGGTAAGA | TTGGCTGGTTTGGTTTCT |
| Lhx2 | CTTGGGTCTCCCGCCTTCC | AGCCCCGGCTCTGCAGTC |

**Supplementary Table S3.** ChIP-qPCR primers for examination of histone modification occupancy in promoters of selected genes.

| Gene | Forward (5'-3') | Reverse (5'-3') |
|---|---|---|
| Pax6 | TCATCTCCCTTGCCTTGC | TTGCTGAAATCCCAACACC |
| Six3 | ACTACCAGGAGGCCGAGAAG | CAGTTCGCGTTTCTTGCTG |
| Sox2 | GCCCTGCAGTACAACTCCAT | TGGAGTGGGAGGAAGAGGTA |
| Nanog | GATTTGTGGGCCTGAAGAAA | GGAAAAAGGGGTTTCCAGAG |
| Oct4 | TTGCCAGCCATTATCATTCA | TATAGAGCTGCTGCGGGATT |
| Zfp42 | GGCATTGGAAATAGCAGA | GGTCCCAGGATGAGAACA |
| Foxa2 | GCTGCGGCTACCTCTCACCT | ACAAGTGCCGCAGTGACGTG |
| Hand1 | GGCAAGGCTGAAAATGAGAC | GATAGCCACTCCCCCTTTTC |
| Gata6 | GGATGAGAACGGTTTCTGGA | TTGTGAACTTGTGGCTCCTG |

**Supplementary Table S4.** ChIP-qPCR primers for examination of H3K9ac occupancy, Sox2 and Pax6 binding in Kat2b knockdown assay.

| Location | Gene | Forward (5'-3') | Reverse (5'-3') |
|---|---|---|---|
| Sox peak | Nrip1[a,b] | CTACCTTCAGCAGCCAAT | TGTGTCCACCTTATTACGAT |
| Sox peak | Lhx2[b] | TCTTATTGCTCTCCGATTCT | CCTTATTGTCTGGTTGTCTG |

| | | | |
|---|---|---|---|
| Sox peak | Lhx2[a] | CCACAGTTGAAGGACCATT | TTGTTGCCGAGACCAGAG |
| Pax6 peak | Pax6[a,c] | CAGTTAAGGTGACAGGAGAG | CAAGAAGAGTCTAGCGTACC |
| Pax6 peak | Lhx2[a,c] | CCAATAACGGCTTCGGAA | CCAGGAGAACCACAGAGA |

Note: a, b, c for occupancy of H3K9ac, Sox2, and Pax6, respectively.
